# Accessory flexor carpi ulnaris: case report and review of the literature

**DOI:** 10.1259/bjrcr.20200010

**Published:** 2020-04-30

**Authors:** Ian Pressney, Bhavin Upadhyay, Sherine Dewlett, Michael Khoo, Anastasia Fotiadou, Asif Saifuddin

**Affiliations:** 1 Department of Radiology, Royal National Orthopaedic Hospital, Stanmore, UK; 2 Department of Paediatrics, Royal National Orthopaedic Hospital, Stanmore, UK

## Abstract

Most of the accessory muscles of the forearm described in the radiology literature are located either in the radial aspect of the forearm or towards the hypothenar eminence. We present an unusual case of an ulnar-sided distal forearm accessory flexor carpi ulnaris muscle presenting as a “pseudotumour“ demonstrated with both ultrasound and MRI, rarely reported in the current surgical and anatomical literature. Given the location and relation to the ulnar nerve towards Guyon’s canal, the accessory muscle may also predispose to distal ulnar nerve entrapment.

## Introduction

Most of the accessory muscles of the forearm described in the radiology literature are located towards the radial aspect of the forearm. These include the accessory flexor digitorum superficialis indicis and flexor carpi radialis brevis vel profundus muscles with variations in the palmaris longus (PL) tendon also commonly described.^1^ Towards the ulnar side of the wrist, hypothenar muscle variations are frequently described with the commonest being an accessory abductor digiti minimi (ADM) muscle.^1^ There are several surgical and cadaveric studies describing accessory muscles in the ulnar aspect of the forearm,^2–4^ but without detailed imaging correlation. To the best of the authors’ knowledge, this is only the second combined ultrasound and MRI description of an accessory flexor carpi ulnaris (FCU) muscle, and the first since 2001.^5^

## Case report; clinical presentation and imaging findings

**Figure 1.**
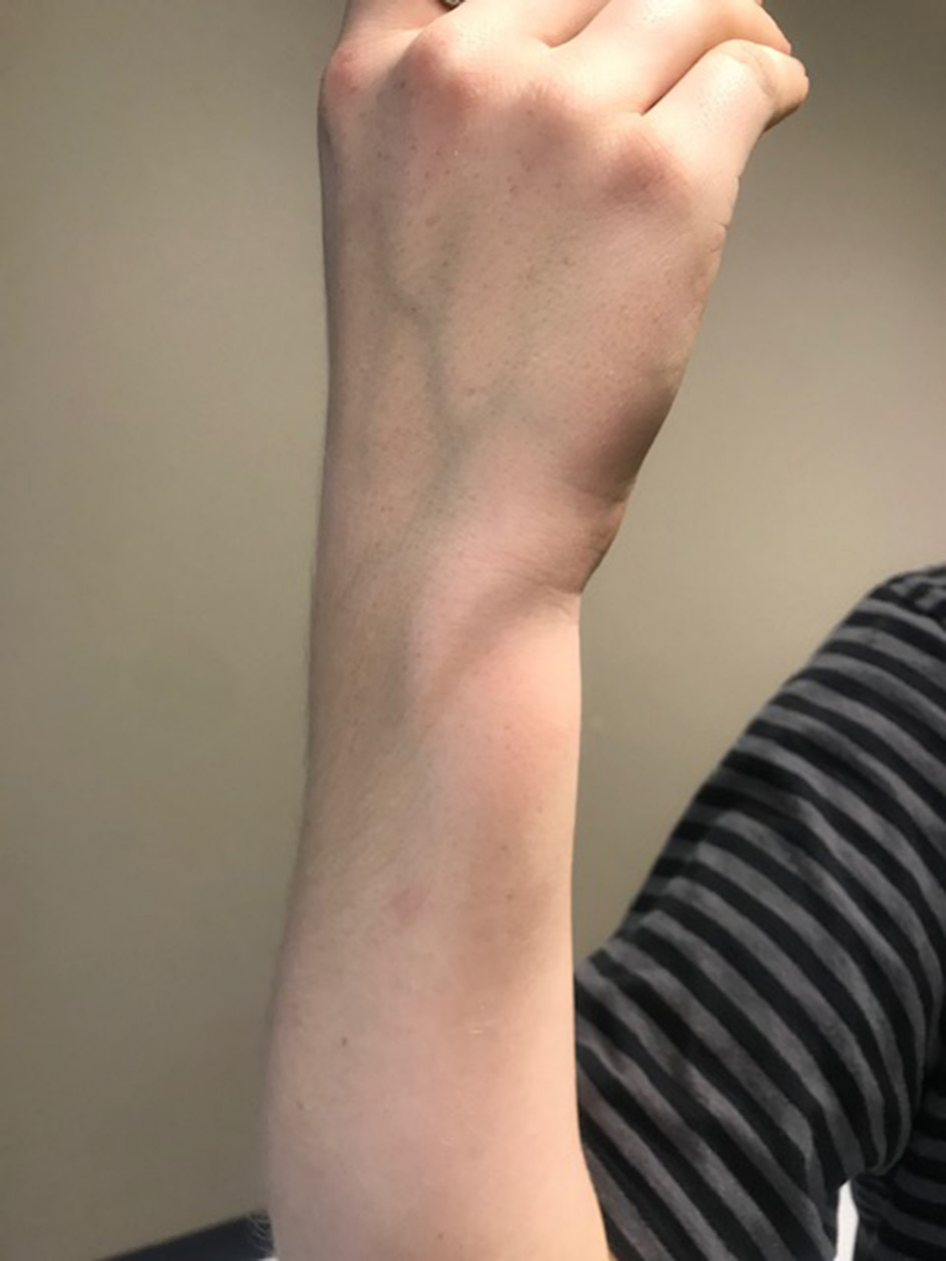
Medical photograph of the “pseudotumour“ towards the ulna aspect right distal forearm.

A 13-year-old girl was referred to our tertiary orthopaedic oncology centre with an ulnar-sided swelling at the volar aspect of the distal right forearm. This was more prominent on flexion of the wrist. Other than the swelling, there were no other clinical symptoms, and in particular no symptoms of ulna nerve compression. On clinical examination, the “pseudotumour“ was smooth and painless on palpation and measured approximately 5 cm in craniocaudal (CC) dimension. (Figure 1) It was located towards the distal forearm on at the ulnar side with prominence particularly elicited during wrist flexion with ulnar deviation. The patient was right hand dominant and a keen rock climber. There was no significant family history of note.

**Figure 2.**
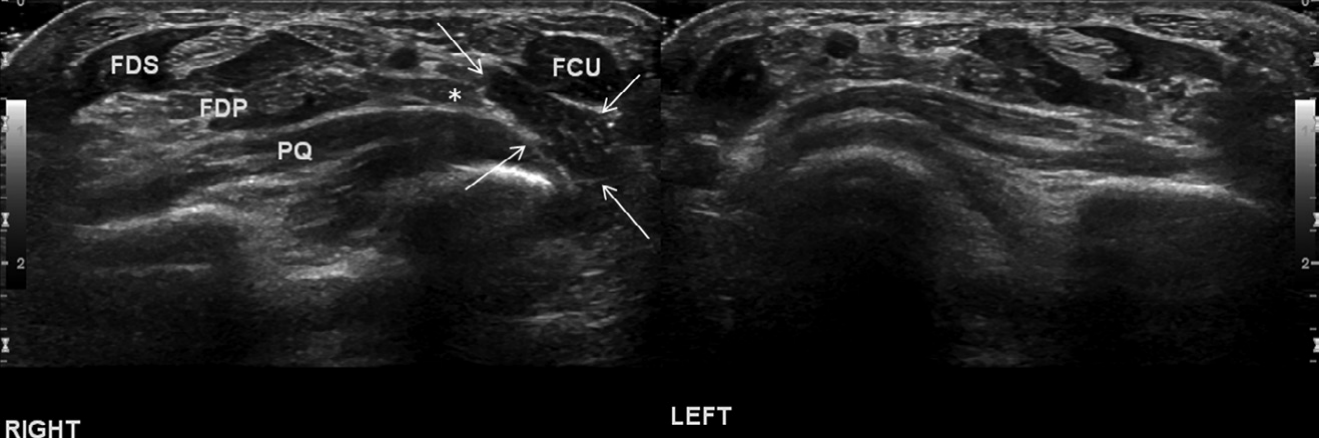
Transverse ultrasound images for “mirror“ comparison of bilateral distal forearms at the level of pronator quadratus muscle demonstrating the accessory muscle bulk deep and separate to normal FCU (white arrows). Note the ulna neurovascular bundle deep to the accessory muscle (asterisk). FCU, flexor carpiulnaris; FDSI, flexor digitorum superficialis.

**Figure 3.**
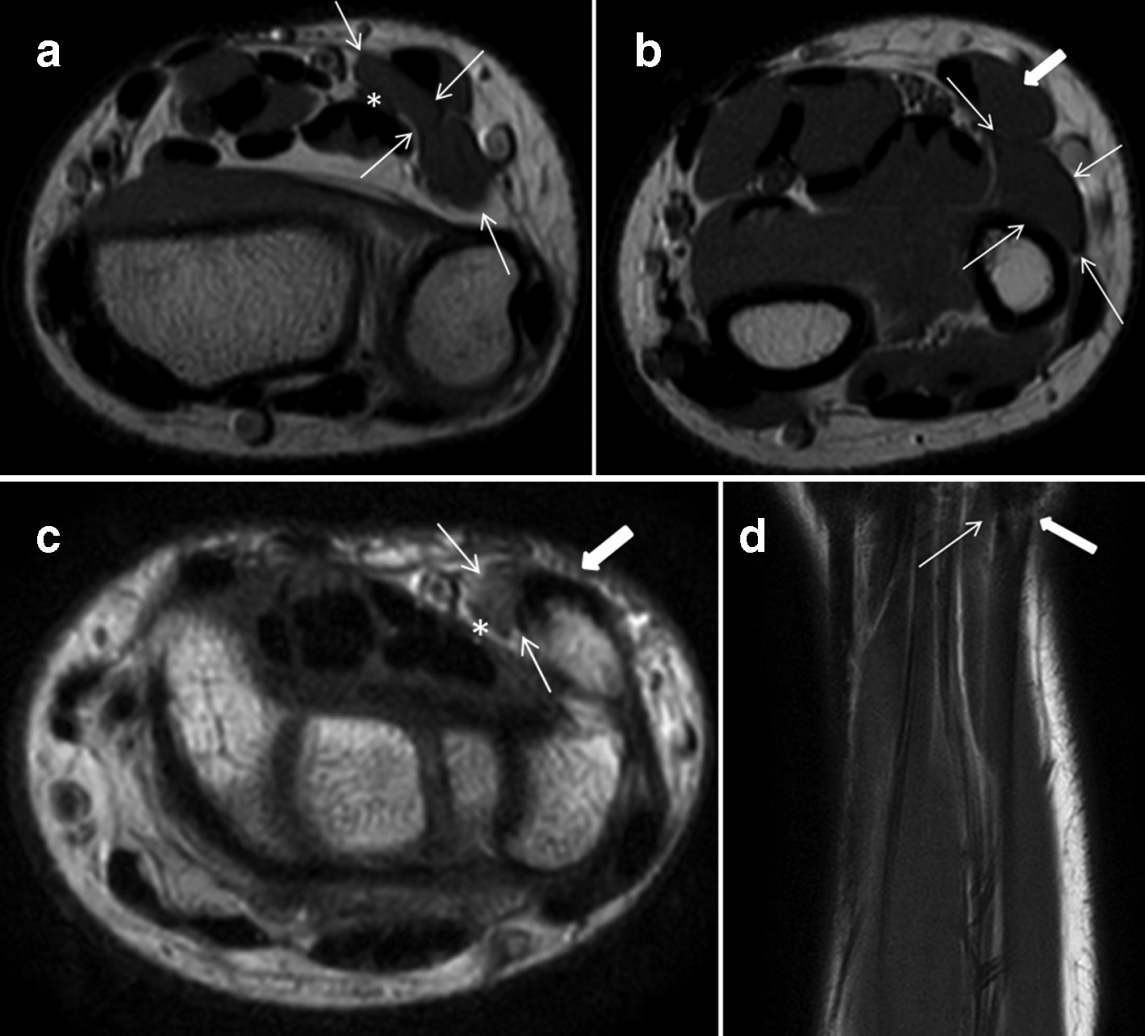
Axial PD weighted images (TR 3314; TE 30) at (a) level just proximal to Guyon’s canal, (b) approximately 3 cm proximal to the radiocarpal joint, (c) at level of pisiform and (d) coronal *T*_1_ weighted (TR 590.52; TE 20) images demonstrating the accessory muscle (white arrows), larger FCU proper (white block arrows), and ulna nerve (asterisks) including their separate tendon insertions to pisiform. Note the normal appearing PL tendon. FCU, flexor carpiulnaris; PD, proton density; PL, palmaris longus; TE, echo time; TR, repetitiontime.

The patient underwent dynamic ultrasound that identified a well-defined hypoechoic structure of identical echotexture to adjacent muscle bellies at the site of clinical concern, just deep to a normal appearing and separate FCU. (Figure 2) It measured in the region of 5 × 1.3 × 0.5 cm in size (CC x transverse x anteroposterior). The ulnar nerve was demonstrated at its deep margin. MRI was undertaken, confirming the ultrasound findings (Figure 3A, B) with a small distal tendon inserting at the pisiform separate to and just radial to the larger FCU tendon insertion (Figure 3C, D). The muscle measured up to 7 cm in CC dimension with its origin at the ulnar aspect of the distal ulna, deep and anterior to the proximal extensor carpi ulnaris (ECU) muscle belly. No intrinsic muscle signal abnormality was demonstrated. The ulnar nerve was demonstrated with normal dimensions and signal intensity, deep to the distal aspect of the accessory muscle belly and proximal to Guyon’s canal.

## Discussion

There are several reports in the literature with regards anomalies of FCU with wide variance in their features. The presence of a supernumerary muscle belly is rare when compared to descriptions of anomalous insertions or small extra tendinous slips. Furthermore, there are inconsistencies in the literature with regard to the nomenclature of the supernumerary muscles that have included digastric FCU,^3^ accessory FCU,^2^ and flexor carpi ulnaris palmaris (FCUP).^6^ Bhardwaj et al recently described a new classification system for supernumerary FCU in the surgical literature in an attempt to consolidate and clearly classify the anomaly, so as the reporting of their incidence and clinical features could be accurately assessed.^4^ The classification was based both on the clinical appearances and probable embryological basis of the anomaly. They separated the different types of anomalies of FCU into three major categories that included; Type I—split tendons, where there is a single muscle with two tendons, Type II—digastric FCU, where two heads of FCU form separate muscle bellies and tendons, and Type III—accessory FCU, where there is an abnormal muscle adjacent to a normal FCU with possible combined features of both FCU and PL. In the current case, there is evidence of an extra muscle in addition to the normal flexor carpi ulnaris, and therefore this is felt to represent a Type III accessory FCU. There are approximately 14 reported cases of Type III FCU anomaly in the anatomy and surgical literature.^2–14^ The embryological basis of the anomaly is thought to be due to the anomalous separation of the flexor blastemal based on the morphology of the reported cases.^4^ Based on the previous case reports and proposed embryological hypothesis, there are other potential anomalies associated with the Type III accessory FCU that include an absent PL, duplication of PL and flexor carpi radialis brevis, in addition to variations of the PL such as insertion onto the flexor retinaculum or median nerve innervation.(Table 1).

**Table 1.** Summary of Type III accessory FCU (modified from Bhardwaj et al^4^)

	Type III accessory FCU
Feature	In addition to normal FCU; An anomalous muscle in forearm with features of FCU and sometimes PL
Possible embryological basis	Due to anomalous separation of flexor blastemal
No. of cases in literature	14
Associated anomalies	Common; absent PL, duplication PL, FCRB
Innervation pattern	Anomalous innervations common

FCRB, flexor carpi radialis brevis; FCU, flexor carpi ulnaris; PL, palmaris longus.

With regards to the imaging literature, there are several MRI illustrations of the accessory FCU in papers by Lemon et al^10^ and Milena at al.^5^ Milena et al^5^ demonstrated a similar accessory muscle on ultrasound and MRI which also had an insertion to pisiform, as in our case. However, the accessory muscle in their case originated more proximally at the lateral edge of the elbow, blending with the ulnar attachment of pronator quadratus. When considering the accessory muscle origin of our case at the volar/ulnar surface of the distal ulna, Mori et al^7^ described four supernumerary muscles of this nature with similar anatomical origin and insertion into the pisiform.

Given the position and location of the accessory muscle, there is potential for ulnar nerve entrapment syndrome with several reports of accessory FCU causing ulnar nerve symptoms that responded well to neurolysis and re-attachment of the distal tendon.^5,8^ In the current case, there were no clinical symptoms of ulnar nerve entrapment. When considering ulnar nerve entrapment syndromes of the upper limb, it should be noted that the accessory anconeus epitrochlearis muscle can cause more proximal ulnar nerve compression in the cubital tunnel, having its origin at the medial epicondyle with insertion to the olecranon. A recent paper found that this accessory muscle accounted for 12 of 142 operated cases of cubital tunnel syndrome.^15^

The ulnar nerve can also be compressed distally at Guyon’s canal. This canal is triangular in shape, with the pisiform forming the medial side of the canal and the deep and superficial volar ligaments forming the deep and superficial boundaries respectively. Within the canal, the ulnar nerve lies medially and the ulnar artery and vein laterally. Reports describe space occupying lesions such as ganglion cyst,^16^ lipoma,^17^ FCU calcific tendonitis^18^ and accessory abductor digiti minimi^19^ causing ulna nerve symptoms, and our case suggests that hypothetically an accessory FCU insertion towards the distal radial aspect of pisiform could potentially cause reduction in canal volume and subsequent nerve compression.

In conclusion, we have described the ultrasound and MRI appearances of the accessory FCU, a rare muscle variant in the distal forearm which should be considered in the differential diagnosis of distal forearm swellings. Detection of FCU variants is potentially important in the assessment of distal ulnar nerve entrapment syndromes.

## Learning points

- Rare muscle variants in the distal forearm should be considered in the differential diagnosis of distal forearm swellings.
- Detection of FCU variants is potentially important in the assessment of distal ulnar nerve entrapment syndromes.

## References

[1] Sookur PA, Naraghi AM, Bleakney RR, Jalan R, Chan O, White LM Accessory muscles: anatomy, symptoms, and radiologic evaluation. Radiographics 2008; 28: 481–99. doi: 10.1148/rg.282075064 18349452

[2] Ang GG, Rozen WM, Vally F, Eizenberg N, Grinsell D Anomalies of the flexor carpi ulnaris: clinical case report and cadaveric study. Clin Anat 2010; 23: 427–30. doi: 10.1002/ca.20952 20196127

[3] Alvin M, Alan N, Leone J, Fredieu JR A unilateral accessory flexor carpi ulnaris muscle observed during cadaveric dissection. Clin Anat 2011; 24: 971–3. doi: 10.1002/ca.21234 21800376

[4] Bhardwaj P, Bhandari L, Sabapathy SR Supernumerary flexor carpi ulnaris--case report and review. Hand Surg 2013; 18: 393–7. doi: 10.1142/S0218810413720222 24156584

[5] López Milena G, Ruiz Santiago F, Chamorro Santos C, Cañadillas Barea L, Milena LG, Santiago RF, Santos CC Forearm soft tissue mass caused by an accessory muscle. Eur Radiol 2001; 11: 1487–9. doi: 10.1007/s003300100827 11519562

[6] Arnold G, Zech M An accessory muscle and additional variants of the forearm. Handchirurgie 1977; 9: 135–6. 608638

[7] Mori M Statistics on the musculature of the Japanese. Okajimas Folia Anat Jpn 1964; 40: 195–300. doi: 10.2535/ofaj1936.40.3_195 14213705

[8] O'Hara JJ, Stone JH Ulnar neuropathy at the wrist associated with aberrant flexor carpi ulnaris insertion. J Hand Surg Am 1988; 13: 370–2. doi: 10.1016/S0363-5023(88)80011-X 3379272

[9] Lim AY, Kumar VP, Hua J, Pereira BP, Pho RW The neuromuscular compartments of the flexor carpi ulnaris. Plast Reconstr Surg 1999; 103: 1046–51. doi: 10.1097/00006534-199903000-00048 10077101

[10] Lemon M, Belcher HJCR An anomalous flexor carpi ulnaris. J Hand Surg Br 2002; 27: 194–7. doi: 10.1054/JHSB.2001.0705 12027500

[11] Georgiev GP, Jelev L, Ovtscharoff WA Unusual combination of muscular and arterial variations in the upper extremity: a case report of a variant palmaris longus and an additional tendinous portion of the flexor carpi ulnaris together with a persistent median artery. Anatomy 2009; 3: 58–61. doi: 10.2399/ana.09.031

[12] Chong SJ, Al-Ani S, Pinto C, Peat B Bilateral flexor carpi radialis brevis and unilateral flexor carpi ulnaris brevis muscle: case report. J Hand Surg Am 2009; 34: 1868–71. doi: 10.1016/j.jhsa.2009.08.002 19897318

[13] Campos D, Nazer MB, Bartholdy LM, Souza PL Accessory flexor carpi ulnaris muscle: a case report of a rare variation in human. J Morphol Sci 2010; 27: 30–1.

[14] Yonguç GN, Cirpan S, Sayhan S, Eyüboğlu C, Güvençer M A variation of flexor Carpi Ulnaris muscle: a case report. J Basic Clin Health Sci 2018; 2: 57–9. doi: 10.30621/jbachs.2018.320

[15] Park I-J, Kim H-M, Lee J-Y, Jeong C, Kang Y, Hwang S, et al Cubital tunnel syndrome caused by Anconeus epitrochlearis muscle. J Korean Neurosurg Soc 2018; 61: 618–24. doi: 10.3340/jkns.2018.0033 30196659 PMC6129750

[16] Kwak K-W, Kim M-S, Chang C-H, Kim S-H Ulnar nerve compression in Guyon's canal by ganglion cyst. J Korean Neurosurg Soc 2011; 49: 139–41. doi: 10.3340/jkns.2011.49.2.139 21519507 PMC3079103

[17] Paget J, Patel N, Manushakian J Ulnar nerve compression in Guyon’s canal: MRI does not always have the answer. JSCR 2013; 1 2pages. 10.1093/jscr/rjs043 PMC3579532 24963936

[18] Yasen S Acute calcific tendinitis of the flexor carpi ulnaris causing acute compressive neuropathy of the ulnar nerve: a case report. J Orthop Surg 2012; 20: 414–6. doi: 10.1177/230949901202000333 23255660

[19] Zeiss J, Jakab E, Khimji T, Imbriglia J The ulnar tunnel at the wrist (Guyon's canal): normal Mr anatomy and variants. AJR Am J Roentgenol 1992; 158: 1081–5. doi: 10.2214/ajr.158.5.1566671 1566671

